# Supplementary material for: Patterns of Intron Gain and Loss in Fungi
Source: PLoS Biol. 2004 Nov 30;2(12):e422. doi: 10.1371/journal.pbio.0020422 (PMC532390; doi:10.1371/journal.pbio.0020422)
Supplement: Table S1 — Also available at http://genes.mit.edu/NielsenEtAl/. (4.3 MB ZIP). [file pbio.0020422.st001.zip › NielsenEtAl/html/333.html]

AN0776.1.NCU03703.1.MG09194.1.FG01871.1


```
 CLUSTAL W (1.82) Multiple Sequence Alignments - Introns Inserted


Sequence 1: NCU03703.1	194 aa
Sequence 2: FG01871.1	185 aa
Sequence 3: MG09194.1	186 aa
Sequence 4: AN0776.1	184 aa
Alignment Length: 194 aa
Number Identitical Residues: 134 aa
Alignment Score (without introns) 5512


MG09194.1 	--------M0VRYGATEIETAKSARARGSYLRVSFKNTRETAQAINGWKLHRAVKFLENV
NCU03703.1	MTESCSKSQ~VRYAATEIAPAKSARSRGSYLRVSFKNTRETAQAINGWKLQRAQKFLENV
FG01871.1 	--------M0VRYAATEIQPAKSARARGAYLRVSFKNTRETAQAINGWKLQRAVTFLENV
AN0776.1  	--------M0VRYAAQDIPAAKSARARGSYLRVSFKNTRETAQAINGMKLQRALAFLENV
          	          ***.* :* .*****:**:****************** **:**  *****

MG09194.1 	QEKKEAVPMRRYAGSTGRCAQG1KQFGVSKARWPTKSAEFLLGLLKNAEANADAKGLDTG
NCU03703.1	LEKKEAVPMRRYAGGTGRAAQG1KQFGVSRARWPAKSAEFLLGLLKNAEANADAKGLDTG
FG01871.1 	KEHKEAVPMRRYAGSTGRTAQG1KQFGVSKARWPIKSAEFLLGLLKNAEANADAKGLDTG
AN0776.1  	KTKTEVVPFRRFASSTGRCAQA1KQWGVARARWPVKSAEFLLDLLKNAEANADTKGLDTG
          	  :.*.**:**:*..*** **. **:**::**** *******.**********:******

MG09194.1 	NLVVKHIQVNQAPKQRRRTYRAHGR0INPYMSCPCHIELILTEQEEAVEKSDAVTDRNEH
NCU03703.1	NLVVKHIQVNQAPKQRRRTYRAHGR0INPYMSNPCHIELILTEADEVVQKSEAVVREEAH
FG01871.1 	ALVVKHIQVNQAPKQRRRTYRAHGR0INPYMSNPCHIELILTEAEEVVQKSDAVA-EREH
AN0776.1  	NLVVKHIQVNQAPKGRRRTYRAHGR0INPYMTNPCHIELILTEGEEEVKK--ATVVERTR
          	 ************* ********** *****: ********** :* *:*  *.. .. :

MG09194.1 	LTSRQRGARVRKALTAA
NCU03703.1	LSSRQRGARVRRAITAA
FG01871.1 	LSSRQRGARLRKAITSA
AN0776.1  	LSSRQRGAQIRKALTSA
          	*:******::*:*:*:*
```
